# A Prospective Cohort Study of the Clinical Predictors of Bacteremia in Under-Five Children With Acute Undifferentiated Fever Attending a Secondary Health Facility in Northwestern Nigeria

**DOI:** 10.3389/fped.2021.730082

**Published:** 2022-02-15

**Authors:** Taofik Oluwaseun Ogunkunle, Timothy Olanrewaju Adedoyin, Samuel Kolade Ernest, Fatimah Hassan-Hanga, Abdulazeez Imam, Rasaq Olaosebikan, Stephen K. Obaro

**Affiliations:** ^1^Department of Paediatrics, University of Ilorin Teaching Hospital, Ilorin, Nigeria; ^2^Department of Paediatrics and Child Health, University of Ilorin/University of Ilorin Teaching Hospital, Ilorin, Nigeria; ^3^Department of Paediatrics and Child Health, Aminu Kano University Teaching Hospital, Kano, Nigeria; ^4^Oxford Centre for Global Health Research, Nuffield Department of Medicine, University of Oxford, Oxford, United Kingdom; ^5^Department of Pharmacology and Experimental Therapeutics, Thomas Jefferson University, Philadelphia, PA, United States; ^6^Division of Pediatric Infectious Diseases, Department of Pediatrics/Department of Microbiology and Pathology, University of Nebraska Medical Center, Omaha, NE, United States

**Keywords:** bacteraemia, acute undifferentiated fever, lymphopenia, *Salmonella*, lethargy, under-five children

## Abstract

**Background:**

Children with acute febrile illness with no localizing signs often receive antibiotics empirically in most resource-poor settings. However, little is known about the burden of bacteremia in this category of patients, and an appraisal is thus warranted. This will guide clinical practice and promote rational antibiotics use.

**Methods:**

We prospectively followed up 140 under-five children who presented with acute undifferentiated fever at the emergency/outpatient pediatric unit of a secondary healthcare facility. Baseline clinical and laboratory information was obtained and documented in a structured questionnaire. We compared baseline characteristics between participants with bacteremia and those without bacteremia. We further fitted a multivariable logistic regression model to identify factors predictive of bacteremia among the cohort.

**Result:**

The prevalence of bacteremia was 17.1%, and *Salmonella* Typhi was the most frequently (40.9%) isolated pathogen. The majority (78.6%) of the study participants were managed as outpatients. The participants who required admission were four times more likely to have bacteremia when compared to those managed as outpatients (AOR 4.08, 95% CI 1.19 to 14.00). There is a four times likelihood of bacteremia (AOR 4.75, 95% CI 1.48 to 15.29) with a fever duration of beyond 7 days. Similarly, participants who were admitted with lethargy were six times more likely to have bacteremia (AOR 6.20, 95% CI 1.15 to 33.44). Other significant predictors were tachypnea and lymphopenia.

**Conclusion:**

Among under-five children with acute undifferentiated fever, longer duration of fever, lethargy, inpatient care, tachypnea, and lymphopenia were the significant predictors of bacteremia.

## Introduction

An acute undifferentiated febrile illness is characterized by a fever of fewer than 2 weeks for which no localizing signs or etiology is found after a full history and physical examination (1, 2). It may be the only symptom of a mild self-limiting illness or antecedent of a serious infection caused by a bacterial pathogen (3, 4). Most cases of acute undifferentiated fever (AUF) are caused by viruses in South and Southeast Asia while malaria parasitemia has frequently been identified in parts of sub-Saharan Africa (5–7).

In most resource-poor settings, polymicrobial infection with viruses, bacteria, and malaria is common and could be clinically indistinguishable (8–10). Although malaria could be rapidly diagnosed in most of these settings, clinicians in these settings are often challenged by the limited access to bacterial culture facilities to aid in accurate diagnosis of bacteremia (11). Also, white blood cell parameters which are available are inadequate to differentiate between malarial, viral, and bacterial pathogens. Hence, children with acute fever are often presumptively prescribed antibiotics, and this could increase the risk of antibiotic resistance (12, 13).

Unlike those with AUF, children with demonstrable focus of infection such as pneumonia and meningitis are rationally prescribed antibiotics according to existing guidelines (14, 15). However, there is currently a dearth of information on the accurate burden of bacteremia and its clinical predictors among children with AUF in most resource-poor settings, especially in sub-Saharan Africa, and this could jeopardize the rational deployment of antibiotics. The Pediatric Early Warning Score (PEWS) and the pediatric quick Sequential Organ Failure Assessment (qSOFA score) have been used to identify patients at risk of poorer outcomes (16–19). The qSOFA have been mostly used to predict mortality from sepsis rather than identifying subjects who had sepsis and needed prompt initiation of antibiotics. Besides, the sensitivity in predicting mortality and severe sepsis has been reportedly poor in the pediatric population (18, 20). Also, the qSOFA included blood pressure measurement, which is often not done routinely in most resource-poor settings. Similarly, the PEWS was designed to identify hospitalized children with heterogeneous disease conditions at risk for clinical deterioration and may not be applicable to children with AUF. Studies identifying the bacterial etiology of childhood AUF as well as its clinical predictors would thus be crucial, and this is the thrust of the current study. This study was initiated on a platform of ongoing surveillance for bacteremia in young children to identify clinical predictors of bacteremia among under-five children with acute undifferentiated fever.

## Materials and Methods

### Study Design and Setting

A prospective cohort study was conducted at the emergency and pediatric outpatient unit of Murtala Mohammed Specialist Hospital (MMSH), Kano. The Department of Pediatrics of the hospital has an outpatient unit, a 30-bed emergency unit and a 50-bed pediatric medical ward. On average, the department conducts about 200–300 pediatric emergency consultations monthly and about 300 outpatient consultations daily. The emergency unit is manned by interns, medical officers, and a consultant and receives pediatric medical emergencies beyond the neonatal age up to the age of 14 years while the outpatient unit is manned by medical officers and a supervising consultant.

### Inclusion and Exclusion Criteria

Subjects were previously healthy under-five children aged from 1 to 59 months with acute undifferentiated fever (i.e., with no localizing symptoms and signs). Consequently, subjects with clinical evidence of meningitis, pneumonia, urinary tract infection, or others with a discernable focus of infection were excluded. Also, subjects known to have sickle cell disease (SCD) or those that have taken antibiotics 48 h prior to recruitment were excluded. Children with SCD mostly have routine antibiotics prophylaxis in the study location.

### Sample Size Determination

The sample size of the study was 140 study participants. This was determined using the standard formula for sample size in an observational study (21). The calculation for this is shown below:


$$\begin{array}{l} n=\frac{{\text{Z}}^{2}pq}{d^{2}} \end{array}$$

where *n* is the desired minimum sample size; *z* is the standard normal deviation set at 1.96, which corresponds to 95% confidence interval, and *p* is the prevalence of bacteremia among children with undifferentiated fever, estimated to be 9% from a previous study (6). Also, *q* = 1– *p* = 1–0.09 = 0.91, while *d* is the tolerable margin of error, with an observed difference of 5% taken as being significant.


$$\begin{array}{l} \text{Therefore},n=\frac{1.96^{2}\times 0.09\times 0.91}{0.05^{2}}=126\;. \end{array}$$

The calculated sample size was 126, and allowing for an attrition rate of 10% (≈13), the minimum sample size estimated was approximately 140.

### Definitions

1. Acute Undifferentiated Fever: Fever of ≥ 38°C for ≤ 14 Days Without Localized or Organ-Specific Clinical Features. Non-localizing Symptoms Include Myalgia, Clear Rhinorrhea, non-Bloody Diarrhoea, Rash, Arthralgia, Headache, Altered Sensorium, and Jaundice (6, 22, 23).

2. Bacteremia: Isolation of at Least one non-Contaminant Bacteria From the Admission Blood Specimen. Coagulase-Negative *Staphylococcus* Species, *Corynebacterium* Species, α- or γ-Hemolytic *Streptococci, Micrococcus* Species, *Bacillus* Species, and *Propionibacterium* Species Were Regarded as Contaminants (24).

### Data Collection

**Figure 1:**
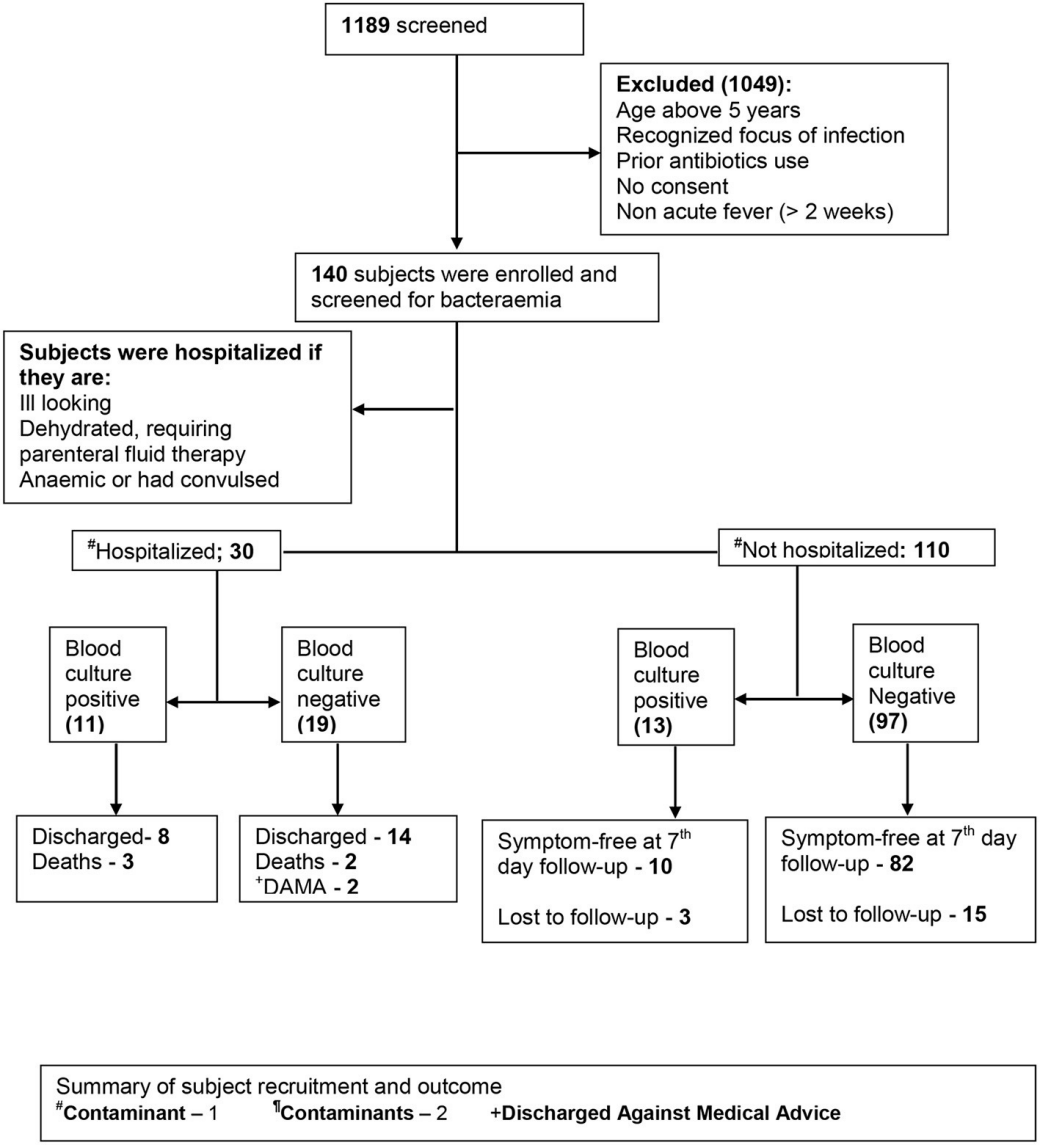
Summary of subject recruitment and outcome.

Not all eligible subjects were enrolled in the study because of insufficient manpower to commit specifically for this study at the enrollment site. Recruitment was conducted during the working hours of weekdays only, excluding public holidays. Subjects fulfilling the preset clinical criteria were recruited during their presentation to the emergency or outpatient unit until the estimated sample size was achieved. This spans a period of 5 months (November 2015 and March 2016). The recruitment was done by a medical officer with at least 2 years of experience of pediatric emergency setting. Clinical history was obtained from the caregivers, and similarly, examination findings were entered in the study pro forma. Each study participant was followed up until outcome: discharge, death, discharge against medical advice, symptom free (outpatient) at 7 day's follow-up, or unknown (outpatients who could not be reached by phone and who did not return for follow-up) (Figure 1).

Complete blood count and bacterial isolation were automated using Swelab and BACTEC™ 9050, respectively. All samples were sent to the laboratory within an hour of collection for incubation, and the vials were incubated in the automated culture system for a maximum of 5 days. A positive reading indicates the presumptive presence of viable microorganisms in the vial, usually within 48 h. Under aseptic conditions, aliquots were obtained from positive vials. Only aerobic culture bottles were used. Aliquots from positive vials were subjected to Gram stain microscopy and subcultured using enriched sheep blood agar or chocolate agar. Inoculated media was incubated under aerobic and 5% CO_2_ conditions at 35°C for 18–24 h (25). Bacteria were identified by a combination of morphology on the growth plate, Gram stain, and standard biochemical tests; *Salmonella* were identified by reaction of the isolate on the Kligler iron agar (KIA)/triple sugar iron agar (TSI); *Streptococcus pneumoniae* were identified by susceptibility to optochin and bile solubility; and the API 20 E system (bioMérieux, France) was used to identify *Klebsiella* and *Proteus* (26, 27).

All ill-looking subjects as assessed by the attending clinician, subjects who convulsed, and those with anemia and dehydration, requiring parenteral fluid therapy, were hospitalized. Subjects were prescribed empirical antibiotics if they were hospitalized and or had tachycardia and tachypnea; otherwise, they were prescribed antipyretics, and their caregivers were reassured. Lumbar puncture was performed on subjects with a recent history of convulsion or symptoms suggestive of meningeal irritation. Follow-up of all non-hospitalized subjects was performed by telephone contact, and the parents were asked to return with the subjects for further assessment within 48 h. Subjects managed as outpatients whose culture result became positive within 48 h were contacted as soon as possible and commenced on antibiotics.

### Exposure Variables

We elicited most of our exposure variables by direct questioning. We derived some other variables; for example, high-grade fever was defined as a temperature > 40^o^C (28). We also classified participants as having tachypnea based on their respiratory rate using the World Health Organization definitions: >60 breaths per minute for children aged below 2 months, >50 breaths per minute for children aged 2–11 months, and >40 breaths per minute for children aged 12–59 months (29). Tachycardia was also defined based on the standard values for age: >160 beats per minute for infants <1 year, >150 beats per minute for those between 1 and 2 years, and >140 beats per minute for those between 2 and 5 years (30). Other laboratory variables were also defined using standard values. Severe anemia was defined as a packed cell value of <18% and thrombocytopenia as a platelet count below 150,000 cells/mm^3^ (31). Lymphopenia was defined as a lymphocyte count <3 × 10^9^/L, neutropenia as a neutrophil count <1 × 10^9^/L, and leukocytosis as a white cell count >15 × 10^9^/L (32).

### Outcome Variable

This was defined as the occurrence of bacteremia.

### Data Analysis

We provided participant summary statistics using the median and interquartile range for non-normally distributed data and frequencies with percentages for categorical data. We compared proportions using chi-square, and when the expected cell count was <5, Fischer's exact test was used. We also compared medians using the Man–Whitney *U*-test. Statistical significance was set at *p* < 0.05 for our univariable analysis. Significant predictors (*p* < 0.2) on univariable analyses were placed in a maximal logistic regression model. The least significant predictor (with *p* > 0.05) was dropped using backwards stepwise regression until the final model, which had only significant predictors. The coefficients of the final model were exponentiated to derive adjusted odds ratios (AORs) and their corresponding 95% confidence intervals (CIs). All statistical analyses were performed using STATA version 16.1 (StataCorp, 2019, Stata Statistical Software: Release 16, College Station, StataCorp LP, TX).

## Results

Between November 2015 and March 2016, 140 under-five children who presented with acute undifferentiated fever were recruited into this study. Of this number, we identified 24 (17.1%) with a positive blood culture and identified an organism in 22 (15.7%) of them. *Salmonella* Typhi was the most frequently cultured organism (40.9% of isolates; Table 1), and this was followed by non-Typhi *Salmonella* (31.8% of isolates; Table 1). Co-trimoxazole had the highest resistance among antibiotics as 77.8% and 85.7% of *Salmonella* Typhi and non-Typhi *Salmonella* were resistant to the antibiotic, respectively (Table 1).

**Table 1:** Antibiogram showing relative frequencies of organisms grown on culture and their different sensitivity profiles.

**Organism type**	**Frequency (%)**	**Sensitivity**	**Co-trim (%)**	**Aug (%)**	**Cefo (%)**	**Genta (%)**	**Cipro (%)**	**Ceftri (%)**
Salmonella Typhi	9 (40.9)	Resistant	7 (77.8)	1 (11.1)	2 (22.2)	0 (0.0)	1 (11.1)	1 (11.1)
		Sensitive	2 (22.2)	7 (77.8)	6 (66.7)	9 (100.0)	8 (88.9)	8 (88.9)
		Not tested	0 (0.0)	1 (11.1)	1 (11.1)	0 (0.0)	0 (0.0)	0 (0.0)
Non-Typhi Salmonella	7 (31.8)	Resistant	6 (85.7)	2 (28.6)	0 (0.0)	0 (0.0)	0 (0.0)	0 (0.0)
		Sensitive	1 (14.2)	4 (57.1)	6 (66.7)	7 (100.0)	7 (100.0)	7 (100.0)
		Not tested	0 (0.0)	1 (14.3)	1 (11.1)	0 (0.0)	0 (0.0)	0 (0.0)
*Streptococcus pneumoniae*	2 (9.0)	Resistant	1 (50.0)	0 (0.0)	0 (0.0)	1 (50.0)	0 (0.0)	0 (0.0)
		Sensitive	1 (50.0)	2 (100.0)	1 (50.0)	1 (50.0)	1 (50.0)	1 (50.0)
		Not tested	0 (0.0)	0 (0.0)	1 (50.0)	0 (0.0)	1 (50.0)	1 (50.0)
*Salmonella* species	1 (4.6)	Resistant	0 (0.0)	1 (100.0)	0 (0.0)	0 (0.0)	1 (100.0)	0 (0.0)
		Sensitive	1 (100.0)	0 (0.0)	1 (100.0)	1 (100.0)	0(0.0)	1 (100.0)
		Not tested	0 (0.0)	0 (0.0)	0 (0.0)	0 (0.0)	0 (0.0)	0 (0.0)
*Klebsiella* specie	1 (4.6)	Resistant	0 (0.0)	0 (0.0)	0 (0.0)	0 (0.0)	0 (0.0)	0 (0.0)
		Sensitive	1 (100.0)	1 (100.0)	1 (100.0)	1 (100.0)	1 (100.0)	1 (100.0)
		Not tested	0 (0.0)	0 (0.0)	0 (0.0)	0 (0.0)	0 (0.0)	0 (0.0)
*Proteus mirabilis*	1 (4.6)	Resistant	1 (100.0)	0 (0.0)	0 (0.0)	0 (0.0)	0 (0.0)	0 (0.0)
		Sensitive	0 (0.0)	1 (100.0)	1 (100.0)	1 (100.0)	1 (100.0)	1 (100.0)
		Not tested	0 (0.0)	0 (0.0)	0 (0.0)	0 (0.0)	0 (0.0)	0 (0.0)
*Stenotrophomonas maltophilia*	1 (4.6)	Resistant	0(0.0)	0 (0.0)	0 (0.0)	0 (0.0)	0 (0.0)	0 (0.0)
		Sensitive	1 (100.0)	1 (100.0)	0 (0.0)	1 (100.0)	1 (100.0)	1 (100.0)
		Not tested	0 (0.0)	0 (0.0)	1 (100.0)	0 (0.0)	0 (0.0)	0 (0.0)
Total	22 (100.0)	Resistant	15 (68.2)	4 (18.2)	2 (9.1)	1 (4.6)	2 (9.1)	1 (4.5)
		Sensitive	7 (31.8)	16 (72.3)	16 (72.3)	21 (95.5)	19 (86.4)	20 (91.0)
		Not tested	0 (0.0)	2 (9.1)	4 (18.2)	0 (0.0)	1 (4.5)	1 (4.5)

*Yellow, Gram-negative organism; Blue, Gram-positive organism; Red, Resistance of organism in >70% of species; Brown, Resistance of organism between 50 and 70% of species. Cotrim, Co-trimoxazole; Aug, Amoxicillin-clavulanate; Cefo, Cefotaxime; Genta, Gentamicin; Cipro, Ciprofloxacin; Ceftri, Ceftriaxone*.

### Participant Outcomes

Thirty enrollees (22.4%) were hospitalized while 110 (78.6%) were managed as outpatients. Ninety-two (83.7%) outpatients were identified as symptom free after a 7-day follow-up, while 18 (16.4%) were lost to follow-up. Among the inpatients, 22 (73.3%) were discharged, 3 (10.0%) were discharged against medical advice, and 5 (16.7%) died while on admission.

### Participant Sociodemographic and Clinical Characteristics

**Figure 2:**
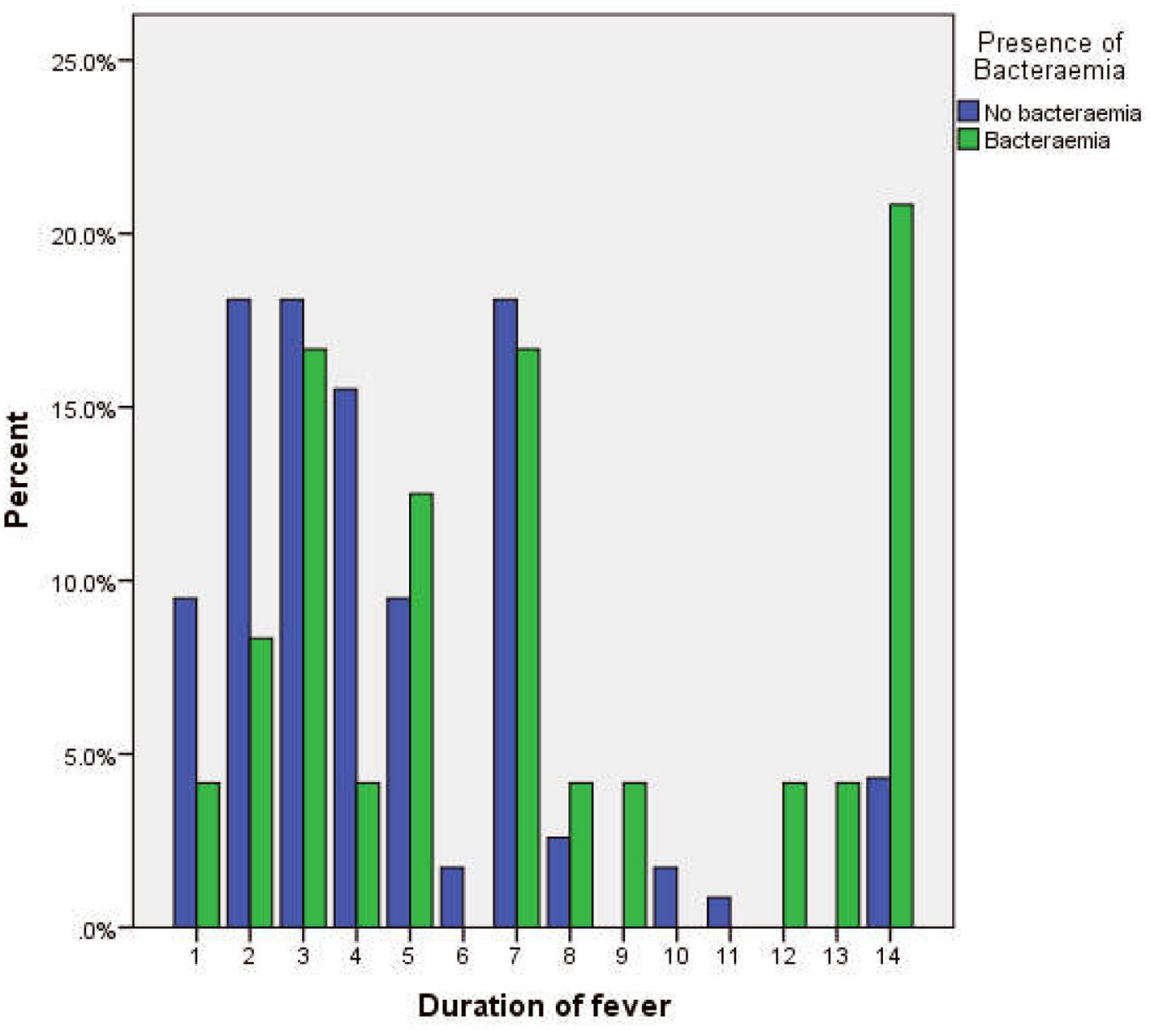
Cluster bar chart showing the relationship between the duration of fever and the occurrence of bacteremia.

There were more subjects with fever beyond 7 days in the group that had bacteremia (Figure 2). The median duration of fever was significantly higher in the bacteremia group (7.0 days) when compared to the group without bacteremia (4 days, *p* = 0.005, Table 2). Similarly, bacteremia occurred more frequently among children admitted as inpatients (*p* = 0.001, Table 2) and presented clinically with a history of lethargy, poor feeding, diarrhea, and abdominal pain (*p* = 0.018, 0.087, 0.070, and 0.078, respectively, Table 2). There was no significant difference in gender, age distribution, or clinical histories of rhinorrhea, vomiting, convulsions, fast breathing, and abdominal distension between the two groups.

**Table 2:** Participant's sociodemographic characteristics and clinical history.

**Variable**	**Bacteremia (%)**,	**No bacteremia**	***p*-value**	**Total (%)**,
	***n* = 24**	**(%), *n* = 116**		***n* = 140**
**Age (months)**			
≤ 12	4 (16.7)	21 (18.1)	0.445	25 (17.9)
13 to 24	6 (25.0)	43 (37.1)		49 (35.0)
25 to 59	14 (58.3)	52 (44.8)		66 (44.7)
**Gender**			
Male	11 (45.8)	66 (56.9)	0.321	77 (55.0)
Female	13 (54.2)	50 (43.1)		63 (45.0)
**Median duration of**	7 (3.0–12.0)	4 (2.0–7.0)	**0.005**	4 (2.0–7.0)
fever*(IQR)	days	days		days
**Duration of fever**			
<7 days	11 (45.8)	84 (72.4)	**0.011**	95 (67.9)
≥7 to ≤ 14 days	13 (54.2)	32 (27.6)		45 (32.1)
**Hospitalization**			
No	13 (54.2)	97 (83.6)	**0.001**	110 (78.6)
Yes	11 (45.8)	19 (16.4)		30 (21.4)
**Rhinorrhea**			
Yes	11 (45.8)	56 (48.8)	0.827	67 (47.9)
No	13 (54.2)	60 (41.2)		73 (52.1)
**Poor feeding^&^**			
Yes	4 (16.7)	40 (34.5)	**0.087**	44 (31.4)
No	20 (83.3)	76 (65.5)		96 (68.6)
**Diarrhea**			
Yes	13 (54.2)	40 (34.5)	**0.070**	53 (37.9)
No	11 (45.8)	76 (65.5)		87 (62.1)
**Vomiting**			
Yes	9 (37.5)	35 (30.2)	0.482	44 (31.4)
No	15 (62.5)	81 (69.8)		96 (68.6)
**Convulsions^&^**			
Yes	2 (8.3)	11 (9.5)	0.860	13 (9.3)
No	22 (91.7)	105 (90.5)		127 (90.7)
**Fast breathing^&^**			
Yes	2 (8.3)	8 (6.9)	0.804	10 (7.1)
No	22 (91.7)	108 (93.1)		130 (92.9)
**Abdominal pain**			
Yes	5 (20.8)	10 (8.6)	**0.078**	15 (10.7)
No	19 (79.2)	106 (91.4)		125 (89.3)
**Abdominal distension^&^**			
Yes	1 (4.2)	3 (2.6)	0.672	4 (2.9)
No	23 (95.8)	113 (97.4)		136 (97.1)
**Lethargy**			
Yes	5 (20.8)	7 (6.0)	**0.018**	12 (8.6)
No	19 (79.2)	109 (94.0)		128 (91.4)
**Dehydration^&^**			
Yes	2 (8.3)	7 (6.0)	0.652	9 (6.4)
No	22 (91.7)	109 (94.0)		131 (93.6)

& *Fischer's exact test performed.*

*Bold value means statistically significant variables*.

### Participant Clinical Examination and Laboratory Findings

A significantly higher proportion of participants in the bacteremia group (25.0%) presented with a high-grade fever when compared to those without bacteremia (7.8%, *p* = 0.013, Table 3). Similarly, a significantly greater proportion of the bacteremia group presented with tachycardia (*p* = 0.015), tachypnea (*p* = 0.01), and lymphopenia (*p* = 0.036) and had fever for longer than 7 days (*p* = 0.011). The distribution of the white cell count also differed across both groups (*p* = 0.018, Table 3). There were no significant group differences in thrombocytopenia, severe anemia, and neutropenia.

**Table 3:** Participants' clinical examination and laboratory findings.

**Variable**	**Bacteremia (%)**,	**No bacteremia**	***p*-value**	**Total (%)**,
	***n* = 24**	**(%), *n* = 116**		***n* = 140**
**Fever grade**				
High grade	6 (25.0)	9 (7.8)	**0.013**	15 (10.7)
Low and moderate grade	18 (75.0)	107 (92.2)		125 (89.3)
**Tachycardia**				
Absent	11 (45.8)	83 (71.6)	**0.015**	94 (67.1)
Present	13 (54.2)	33 (28.4)		46 (32.9)
**Tachypnea**				
Absent	11 (45.8)	84 (72.4)	**0.011**	95 (67.9)
Present	13 (54.2)	32 (27.6)		45 (32.1)
Thrombocytopenia^*^				
Absent	15 (75.0)	76 (83.5)	0.370	91 (82.0)
Present	5 (25.0)	15 (16.5)		20 (18.0)
Severe anemia^*^				
Absent	21 (95.5)	90 (93.8)	0.760	111 (94.1)
Present	1 (4.5)	6 (6.2)		7 (5.9)
Lymphopenia^*^				
Absent	10 (45.5)	67 (69.1)	**0.036**	77 (64.7)
Present	12 (54.5)	30 (30.9)		42 (35.3)
Neutropenia^*^				
Absent	20 (90.9)	87(89.7)	0.864	107 (89.9)
Present	2 (9.1)	10 (10.3)		12 (10.1)
White cell count^*^				
Normal	18 (81.8)	64 (66.0)	**0.018**	82 (68.9)
Leukocytosis	0 (0.0)	25 (25.8)		25 (21.0)
Leukopenia	4 (18.2)	8 (8.2)		12 (10.1)

* *Missing data, not all participants had blood samples taken.*

*Bold value means statistically significant variables*.

### Clinical Predictors of Bacteremia

Subjects are four times more likely to have bacteremia when they have fever beyond 7 days (AOR 4.75, 95% CI 1.48 to 15.29, Table 4). Participants who were admitted with lethargy were 6.2 times more likely to have bacteremia (AOR 6.20, 95% CI −1.15 to 33.44, Table 4). Those admitted as inpatients were also four times more likely to have bacteremia when compared to outpatients (AOR 4.62, 95% CI 1.45 to 14.72, Table 4).

**Table 4:** Risk predictors for bacteremia.

**Variables**	**AOR (95% CI)**	***p*-value**
**Duration of fever**		
≥7 to ≤ 14 days	4.75 (1.48–15.29)	0.009
<7 days	1.00	
**Tachypnea**		
Yes	6.27 (1.88–20.87)	0.003
No	1.00	
**Hospitalization**		
Yes	4.62 (1.45–14.72)	0.010
No	1.00	
**Lymphopenia**		
Yes	4.14 (1.31–13.11)	0.016
No	1.00	
**Lethargy**		
Yes	6.20 (1.15–33.44)	0.034
No	1.00	

## Discussion

We investigated the burden and factors predictive of bacteremia among under-five children with acute undifferentiated fever seen at the emergency unit in northwestern Nigeria. We found that one in six participants had bacteremia, majority from Gram-negative enteric pathogens. Inpatient care, longer duration of fever, lethargy, tachypnea, and lymphopenia were all associated with the increased odds of bacteremia.

The prevalence of bacteremia among under-five children with acute undifferentiated fever was 17.1%. This is similar to the rate reported among children with similar characteristics in Benin, Nigeria (5). However, a higher prevalence rate (25.7%) was reported among Indian children (33). The diagnosis of bacteremia in the current study was based only on blood culture as opposed to the Indian study where the Widal test, a serologic test fraught with high false-positive rates, complemented blood culture in the diagnosis of *Salmonella* infection; this may have overestimated the burden of bacteremia (34). In contrast, the prevalence of bacteremia was significantly lower (1.4%) in a previous study conducted in Brazil with a similar study population to ours (35). The higher positive culture rate found in our study could be because of the utilization of a more sensitive automated culturing system, unlike the Brazilian study where the conventional manual bacteriological culture method was used.

Gram-negative pathogens were the predominant cause of bacteremia among our cohort, accounting for over 80% of the isolates. The preponderance of Gram-negative pathogens in childhood bacteremia had been described previously in Northern Nigeria and in other low- and middle-income countries (5, 7, 33, 36). The preponderance of enteric Gram-negative pathogens in the study location, like in most resource-poor settings, could be related to the high presence of predisposing factors such as high level of poverty, limited access to clean water, poor sanitation, and malnutrition (37). In contrast, some studies have reported predominantly Gram-positive pathogens, especially *Staphylococcus aureus* among febrile children (38–40). While this difference might reflect geographical variations of bloodstream bacterial pathogens, the characteristics of the study participants and the study design could also account for the differences in the organisms isolated. The current study is prospective; hence, it has very minimal selection bias, unlike the previous studies, which were retrospective in design. Also, our subjects are those with acute undifferentiated febrile illnesses and are also devoid of peculiar risk factors such as HIV infection that could predispose to *Staphylococcus* aureus infection (41).

*Salmonella* infection accounted for most of the cases, consistent with the previous reports from the same study location (27, 36). Bacteremia caused by *Salmonella* is most prevalent in the dry season (October–March), and the conduct of the present study during this peak period may have accounted for the predominance of the organism (36). Meningococcal bacteremia was not found in the current study despite the timing of the study, which coincided with the seasonal peak of *Neisseria meningitides*. This observation may, however, not reflect the burden of meningococcal bacteremia in the study location because subjects with overt clinical features of meningitis such as neck stiffness and abnormal tone were excluded from the current study.

*In vitro* antibiotic susceptibility testing revealed a high prevalence of co-trimoxazole resistance, especially by *Salmonella* infection, but with high sensitivity to gentamicin, ciprofloxacin, ceftriaxone, and amoxicillin-clavulanate. A previous multicenter study in Northern Nigeria has also shown high resistance of non-typhoidal *Salmonella* and *Salmonella* Typhi to co-trimoxazole (36). This finding has some implications; first, amoxicillin-clavulanate would seem a rational antibiotic on an outpatient basis, but it is quite expensive compared to co-trimoxazole. Second, ciprofloxacin is cheap and available in both oral and parenteral forms and could be used in both inpatient and outpatient settings, but the widespread use of ciprofloxacin has been associated with a high incidence of extended-spectrum β-lactamase enzyme-producing pathogen, hence increasing antibiotic resistance (42). Third, like ciprofloxacin, ceftriaxone is a broad-spectrum antibiotics, and its use as a first-line drug could also promote resistance. Also, it is expensive and available only in a parenteral formulation and would be less affordable in resource-poor settings where the majority have no form of health insurance.

In this study, the duration of fever rather than the degree of fever was associated with increased odds of bacteremia, and this is similar to previous studies (6, 43). It was further identified that subjects were four (4) times more likely to have bacteremia if fever was present beyond 7 days. Given that the majority of subjects without bacteremia had fever for <7 days, which may have been due to undiagnosed viral illnesses, the increased likelihood of bacteremia after 7 days may thus represent a bacterial superimposition. Other studies have also failed to demonstrate a correlation between the degree of fever and positive bacterial culture (44, 45). Fever results from a cytokine-mediated elevation of the hypothalamic set point and is perhaps the earliest systemic feature of microbial invasion of the bloodstream (46). An increasing duration of fever could therefore reflect an intense bacterial multiplication and an increased tendency of positive culture as obtained in our study. Furthermore, participants who required hospitalization were four times more likely to have bacteremia when compared to outpatients, while those admitted with lethargy and fast breathing were six times more likely to have bacteremia, and this is consistent with a previous study (35). Febrile children presenting with lethargy and fast breathing often appear ill, necessitating inpatient care. In this study, a subjective assessment of “ill/toxic look” made by the attending emergency clinician was one of the eligibility criteria for hospitalization; the discriminatory power of the criteria thus gave it credibility and could thus be adopted in triaging febrile children in other resource-poor settings where facilities and resources for inpatient care are suboptimal.

Among all hematological parameters, a low lymphocyte count remained predictive of bacteremia, and participants with lymphopenia were four times more likely to have bacteremia compared to those who did not have lymphopenia. This finding could be due to the predominance of *Salmonella* species among the organisms isolated. Previous studies, including a controlled human infection model, have consistently demonstrated an association between *Salmonella* infections and lymphopenia (47, 48).

## Strengths and Limitations

We have described the burden and the predictors of bacteremia among under-five children with acute undifferentiated fever. Such children pose a diagnostic challenge, and clinicians are often in a dilemma whether to use antibiotics empirically or not, unlike previous studies on bacteremia conducted among children with focal signs of infection, e.g., meningitis or pneumonia, in whom the deployment of empirical antibiotics is justified. In addition, our study design was prospective, and this enabled the recruitment and the collection of data from all our participants using preset clinical criteria, thus minimizing selection bias as observed in a previous study (7).

However, about 13% of the study participants were lost to follow-up, and hence, the mortality rate as presented may not reflect the actual death attributable to bacteremia among the study participants. In addition, the potential impact of the pathogens causing bacteremia on the identified predictors could not be assessed due to the preponderance of *Salmonella* among the isolates. Therefore, this picture may not apply in other settings where pneumococcal or other forms of bacteremia are most prevalent.

## Conclusions

We have shown that one in six under-five children presenting to a typical emergency unit in Northwestern Nigeria with AUF had bacteremia, and this was mainly due to *Salmonella*. Participants who required hospitalization, those that presented with lethargy, had fever beyond 7 days, had tachypnea, or had lymphocytes counts <3 × 10^9^/L, were at increased odds of bacteremia. We, therefore, recommend that in settings similar to ours, the presence of these clinical predictors could form the for a rational ordering of blood culture. Also, where facilities for blood cultures are not available, febrile under-five children without focal signs but possessing the highlighted clinical features should be treated empirically for bacteremia using the most sensitive antibiotics.

## Data Availability Statement

The raw data supporting the conclusions of this article will be made available by the authors, without undue reservation.

## Ethics Statement

The study was reviewed and approved by Ethics Committees of the University of Nebraska Medical Center, Aminu Kano Teaching Hospital and the Kano State Hospital Management Board. Written informed consent to participate in this study was provided by the participant's legal guardian/next of kin.

## Author Contributions

TO conceived the study, recruited, and obtained clinical data. TO, TA, SE, and FH-H designed the study protocol. AI analyzed the data. TO and AI prepared the first draft of the manuscript. TO, RO, and SO are guarantors for the paper. All authors contributed to the first major revision of the manuscript, final manuscript revisions, and approval of the final version.

## Funding

Research reported in this publication was supported in part by the National Institute of Allergy and Infectious Diseases of the National Institutes of Health (Grant Number. R01AI097493); the Bill & Melinda Gates Foundation (Grant Number. OPP1034619); internal grants from the University of Nebraska Medical Center.

## Conflict of Interest

The authors declare that the research was conducted in the absence of any commercial or financial relationships that could be construed as a potential conflict of interest.

## Publisher's Note

All claims expressed in this article are solely those of the authors and do not necessarily represent those of their affiliated organizations, or those of the publisher, the editors and the reviewers. Any product that may be evaluated in this article, or claim that may be made by its manufacturer, is not guaranteed or endorsed by the publisher.

## Abbreviations

AOR: Adjusted odds ratio
IQR: Interquartile range
AUF: Acute undifferentiated fever
EPU: Emergency pediatric unit
MMSH: Murtala Muhammad Specialist Hospital.
